# Medicine

**Published:** 1898-06

**Authors:** G. Parker


### Progress of the Medical Sciences.

### MEDICINE.

Since we find from the Registrar General's reports that 60,000 persons die annually in the United Kingdom from **phthisis** alone, it is clear that there is good reason for the perennial interest shown in the subject. If we cannot yet point to a specific remedy, we have at least a rapidly-diminishing mortality, and are no longer compelled to regard the disease as an incurable one. Nothing is more certain than that great numbers of patients in the earlier stages do regain good health under proper treatment. The manner of the spread of phthisis has had some fresh light thrown on it by the experiments of Arthur Ransome, who shows clearly that Koch's original statement that the bacillus is a pure parasite, multiplying only at or near the temperature of the body, is untrue. Ransome [1] has proved that it often lives as a saprophyte, growing on damp walls in dark, ill-ventilated houses. He used as media potato, wall-paper, and other substances previously soaked in the moisture collected from cellar air, human breath, and from the air of crowded rooms. On these substances the germ grew and multiplied at the ordinary indoor temperature of the air. Hence he naturally expresses a fear that lecture-rooms, churches,

[1] *Lancet*, 1898, i. 15.

theatres, and assembly-rooms, when dark and ill-ventilated, are but gardens for the cultivation of the bacillus. Others had grown the bacillus at low temperatures, but usually on some specialised medium.

Flügge,[1] however, shows that much of the fear of **infection** from the dust of dried sputum is ill-founded. Not only does the tubercle bacillus fail to float along as dust so readily as other microbes, such as the staphylococcus, but he states that almost all experimental infection of animals by the inhalation of dry dust has failed, though the dust from the rooms of patients readily induces the disease if inserted into the peritoneal cavity. From any less accurate observer such statements would be almost incredible; but even he allows that the finely-divided fluid sputum produced by coughing can be carried along in weak currents of air, and constitutes a source of danger. Thus light, ventilation, and dry air are still to be regarded as hindrances to the spread of the germs.

Gardiner's[2] researches on the **immunity** found **at high altitudes** are also helpful on the subject. First of all, he recognises that a very few cases of phthisis do arise there and are rapidly fatal: but there is no general spread of the disease even when many invalids are collected together, as in Colorado city; yet fresh bacilli grow there very well in cultivation tubes, but the dust from infected rooms is not poisonous when inoculated. The bacilli are therefore, rapidly killed by the dry and sunny climate and rarefied air. Another source of infection, too, is absent, for the cattle are almost exempt from tuberculosis and the milk is in consequence pure. But besides this absence of external infection, there must be something in the individual residents which increases their resistance and enables phthisical immigrants to recover. This he ascribes to several factors—the increased activity of expansion of the lungs, the stimulation of the nervous system by the climate, the dryness of the soil, and the increase of the red corpuscles in the blood, which is constantly found at high altitudes. It may be replied that the increase of red corpuscles is only a compensatory effort to obtain a normal amount of oxygen from the rarefied air, and if no more oxygen is carried in there can be no increase in the $CO_2$ given off. Still, there may be some indirect action unfavourable to the bacilli and helpful to the tissues in this condition or an increased excretion of some other product besides $CO_2$, though we know nothing of the chemical toxin formed or of the elements of the blood which neutralise it. In connection with this resistance of the blood to various toxins, reference may be made to the part played by the granules in the plasma, which are probably akin to those seen in the eosinophile leucocytes.[3] If both leucocytes and the

[1] *Deutsche med. Wchnschr.*, 1897, xxiii. 665; abstract in *Brit. M. J.*, 1898, i. Epitome, p. 13.

[2] *Am. J. M. Sc.*, 1898, cxv. 131.

[3] *Johns Hopkins Hosp. Bull.*, 1897, viii. 246.

free granules are removed from typhoid serum no "clumping" of bacilli takes place, and, indeed, the granules seem to take an active part in the attack, while the cells later on absorb the paralysed microbes. These results, however, merely confirm Kanthack's assertions as to the destruction of anthrax in the frog, and it is uncertain how far they apply to tubercle.

Whatever is the real cause of this immunity at altitudes above 5,000 feet, there is a large amount of evidence that remarkable success has been obtained by the **open-air treatment** of phthisis at lower levels, whether this arises from the prevention of associated infections or from the stimulus to the general health. One of the most interesting attempts has been that of Burton-Fanning,[1] who has treated a number of hospital patients in a sanatorium at Cromer, and finds better results from it than are given by any other method. His patients sleep with open windows, and spend the whole day in open huts or shelters on the lawn. They have plenty of rugs and hot bottles, and are carefully watched for the first week or two. Those whose temperature does not fall below 99.5° in the morning are not allowed out of bed, but those with only evening temperatures show gradual improvement under the treatment. Absolute rest in the shelters is enforced on all whose pulses are at all weak, and this he looks on as an essential part of the treatment; while a liberal diet is allowed to all, to which the rapid increase of appetite in the open air soon does justice. What is particularly noteworthy under such conditions is that greater improvement takes place in the cooler months than in summer, and that the treatment can be carried on in winter in the English climate, in spite of its constant changes and dampness. In Germany, indeed, proof is not wanting that successful out-of-door treatment is attainable, as at Nordrach and Falkenstein, at low levels and in treacherous climates; but, as the *Lancet* remarks,[2] the fact that the method can be used with advantage in our fog-bound island ought to lead to very marked results. If it is possible to dwell in the open air and yet to remain in one's native country, as these experiments show, there is a wide field for sanatoria, which may offer to phthisical patients far better means of recovery than are now within the reach of the inhabitants of our crowded cities. Since it is calculated that homes at present exist for only about one in two hundred of phthisical patients in the hospital classes, a great work is to be done if we aim at reaching the 30 per cent. of absolute or relative cures which are said to take place at Falkenstein, or even the 21 to 24 per cent. which are given at some of the high-altitude sanatoria.

The results of Koch's new **R tuberculin** seem to be generally unsatisfactory. One observer after another announces its failure, if not real dangers from its use. J. Dutton Steele[3] summed up

[1] *Lancet*, 1898, i. 630. [2] *Ibid.*, 942. [3] *Internat. M. Mag.*, 1897, vi. 756.

the reports to the end of the year by saying that the results on existing tubercular lesions are not yet definitely certain, but in lupus and some other cases it is valuable. The new product is not more dangerous than the old, if given carefully; but many samples are contaminated, and the increase of doses ordered is far too great and rapid.

On the other hand, **creasote**, when given in large enough quantities, does continue to find favour, and may be combined with climatic and other treatment. The full benefit is not, however, obtained till doses of from 30 to 90 drops are taken thrice daily. Chaumier[1] notices that guaiacol is in no way superior to pure creasote; but he finds that both may derange the digestion, and advocates on this ground **creasotal** in drachm doses. As a matter of fact, creasote simply beaten up in a cup of milk or well diluted in a mixture most often causes marked improvement of digestion. Von Leyden, however, of the Charité Hospital, makes the same complaint against creasote, but finds that creasotal has all its advantages without any drawbacks.[2] Indeed, he claims a specific action for creasotal given in doses of 5 up to 25 drops. His patients, if in an early stage, improved rapidly and steadily, and in some cases complete cures resulted. Creasote has also been found useful in tubercular peritonitis. Thomas[3] is opposed to laparotomy for this disease except as a last resort, since it only effects a cure where the tubercles are in the fibrous stage and is valueless where they are caseous; but he has given with much success enemata of 5 to 30 minims of creasote in olive oil every night, to which a few drops of laudanum may be added. It is interesting to find an ancient remedy, Bishop Berkeley's tar-water, also recommended as an alternative to creasote by Dr. A. James,[4] of the Edinburgh Infirmary; but most of these writers confess that the use of creasote and its allies is after all empirical, or at least only of value by destroying the associated bacteria, and it is therefore worth while remembering that Dr. Kington Fyffe showed definitely some years ago that tubercle bacilli become markedly degenerate in patients who have taken sufficient creasote, and when inoculated into animals produce little effect. Night sweats are often a great difficulty in treatment. Sometimes they are due to a secondary sepsis, sometimes to tubercular action alone. Salter[5] finds that the sweat generally contains large quantities of tuberculin, which is thus eliminated from the system. Hence he is opposed to interfering with it by atropine, camphoric acid, and other drugs. However, we check an eliminative diarrhœa when it becomes too violent for the patient's strength, except perhaps in uræmia and kidney disease, so that his argument may be carried too far.

[1] *Lancet*, 1898, i. 222. [2] Quoted by Seifert, *Lancet*, 1898, i. 960.
[3] *Rev. méd. de la Suisse Rom.*, 1897, xvii. 712; abstract in *Brit. M. J.*, 1898, i. Epitome, p. 15.
[4] *Scottish M. & S. J.*, 1898, ii. 193. [5] *Lancet*, 1898, i. 152.

**Pneumonia** is now recognised to be a process set up by many different bacteria, even when lobar in type, and the difficulties of treatment are increased by our inability to distinguish clinically its varieties and their causes. Thus J. W. Moore[1] recognises, besides those forms due to the diplococcus and the bacillus of Friedländer, special types found in erysipelas, influenza, tuberculosis, and typhoid, as well as those due to actinomycosis, aspergilli, diphtheria, and, we may add, to the organisms of acute rheumatism and small-pox. Besides ordinary septic pneumonia complicating erysipelas, Moore has observed, in migratory pneumonia, patches of erysipelas on the skin, which changed their position as the area of consolidation in the lungs did. The exciting organism here seems to be a streptococcus; in influenzal pneumonia, besides Pfeiffer's bacillus, a similar growth, has been often discovered. A true pneumonia, due to the tubercle bacillus, occurs in cases of acute phthisis in young persons, while in typhoid, besides the frequent occurrence of septic and diplococcal pneumonia, during the depression due to typhoid poison, we find some evidence of occasional pneumonias produced by Eberth's bacillus itself. Generally speaking, the coccus of Friedländer is less virulent than the diplococcus of Fränkel, and this is itself a delicate organism which soon dies out—so much so, indeed, that in pneumonias lasting beyond the normal time we nearly always find some other microbe, such as the streptococcus.

Dürck[2] finds that any of these germs may be present in actually healthy lungs, and that if blown down a tracheal tube they are incapable of causing the disease. If, however, dust be injected, or the animals be first kept warm and then immersed in a cold bath, lobar pneumonia frequently follows such an insufflation. Recent research, too, has shown the presence of the diplococcus not only in the mouths of a few persons, but almost universally. In pneumonia, under favourable conditions, this organism not only multiplies in the lung-cells—that is, outside the body cavity,—but gains entrance to the blood and produces a true septicæmia. Thus Kohn[3] found it in the blood in 9 cases out of 32, and it is noticeable that seven of these were the only fatal ones of the series. On the whole, then, we see that various harmless microbes, when the body resistance is lowered, excite inflammation in the lungs, and even the diplococcus may produce a septicæmia. Thus no hard and fast line can be drawn between "croupous" and "septic" pneumonia. Little reliance has hitherto been placed in the various forms of serum proposed for pneumonia, and this is not surprising if we consider (1) the difficulty of diagnosing the active agent in the disease, (2) the possible existence of a

[1] *Brit. M. J.*, 1898, i. 14.
[2] *Deutsches Arch. f. klin. Med.*, 1896-7, liii. 368.
[3] *Deutsche med. Wchnschr.*, 1897, xxiii. 136; quoted in *Am. J. M. Sc.*, 1897, cxiv. 499.

depressing cause, such as the toxin of typhoid, which keeps open the gate for fresh infection. Still, good results have been obtained in many cases. Washbourn [1] immunised a horse for nine months and obtained a fairly powerful serum, capable of neutralising highly fatal doses of bacilli. Its protective power in early stages of the disease seems definite, but its bactericidal effects *in vitro* are uncertain and its antitoxic ones unknown.

Another serum, or rather antitoxin, from which great things were hoped is that for **tetanus**, and Lambert,[2] in a valuable analysis of 114 cases where it was used, shows that it really has some value, though we are apt to think that mild cases get well with any treatment, and acute cases, *i.e.* those where the incubation period is not more than eight days, or if longer, is followed by very rapid progress,—that these die with or without treatment. In the 114 cases, with a total mortality of 40 per cent., there were 47 acute ones. If we exclude some doubtful cases and those where death took place before the injection had the necessary time to act, the mortality in these acute attacks was 61 per cent. and the recoveries 38. Now, as the mortality with ordinary methods amounts to 80 per cent., we see a clear saving of life equal to about 18 cases in a hundred. Though the antitoxin cannot undo the injury already done, it destroys any toxin circulating in the blood and any more which the bacilli may produce from their habitation near the original wound. The poison appears to be non-albuminous and the most deadly known to science, twenty times as fatal as the same weight of cobra poison and two hundred and fifty times as strong as strychnia. Nor does it seem to be a ferment, but an actual toxin elaborated by the bacilli which remain living near the spot where they originally entered. It is evident that disinfection of the wound is of the highest importance, but Lambert notices that ordinary disinfectants, such as carbolic and perchloride solutions, are inefficient unless mixed with a half per cent. of hydrochloric acid. Caustic potash in a .4 per cent. solution or trichloride of iodine may also be used as well as a thorough cleansing of the spot; and not least, if there is any reason to suspect infection, a preventive injection of the antitoxin is far more reliable than any measures taken after the symptoms have developed.

In most of these infections **prevention** may be carried out either by keeping away the active germ and the associated ones to which it owes its virulence, or by rendering the organism an unsuitable soil for it. Now, what constitutes an unsuitable soil? Apart from antitoxins, we are generally told to fall back on general constitutional measures, cleanliness and tonic treatment, for the latter half of the work. But such phrases imply

[1] *Brit. M. J.*, 1897, ii. 1849. [2] *N. York M. J.*, 1897, lxv. 754.

either ignorance or inaccurate expression. What, for instance, is "debility" but an inadequate diagnosis of a local lesion and its effects? Dirt, too, as Watson Cheyne said, is of no consequence, provided it does not contain germs. Thus Poehl's attempt to discover the causes of inefficient tissue respiration and vulnerability is in the right direction.[1] He thinks that this inefficiency depends on (1) low alkalinity of the blood, (2), defective oxidation, (3), retention of effete products, and (4) intestinal fermentation. An examination of the urine will show which of these factors are present. If the bisodic phosphates are less than half the normal, there is low alkalinity. Defective oxidation is present when the urea-nitrogen is less than nine-tenths of the whole nitrogen excreted, or when the sodium chloride is less than half the urea. Fermentation and auto-intoxication are shown by a great excess of indican, and by the organic sulphates amounting to more than one-tenth of the inorganic. A test in a simple form for the last condition is certainly a desideratum. Pelligrini,[2] from several experiments, thinks that we have a trustworthy index of the degree of toxicity of the urine in the amount of potassium indoxyl sulphate present. To estimate this he takes 4 c.c. of urine and adds slowly one-third of its volume of pure strong sulphuric acid. The tube is cooled by placing it in cold water, and 1.5 c.c. of pure chloroform is then added. The whole is mixed and the chloroform finally allowed to settle. If there is only a normal amount of the sulphate present the chloroform will show a light blue tint, which is darker if the amount is increased. In melancholia and other cases this sign will point to the necessity of correcting some digestive disorder.

G. PARKER.

[1] *Wien. med. Wchnschr.*, 1897, No. 4; abstract in *Brit. M. J.*, 1897, i. Epitome, p. 92.
[2] *Riv. sper. di Freniat.*, 1897, xxii. 114; abstract in *Brit. M. J.*, 1898, i. Epitome, p. 36.
[3] *Ann. Surg.*, 1897, xxvi. 529.

